# Exercise, diet, and cognition in a 4-year randomized controlled trial: Dose-Responses to Exercise Training (DR's EXTRA)

**DOI:** 10.1093/ajcn/nqab018

**Published:** 2021-03-19

**Authors:** Pirjo Komulainen, Jaakko Tuomilehto, Kai Savonen, Reija Männikkö, Maija Hassinen, Timo A Lakka, Tuomo Hänninen, Vesa Kiviniemi, David R Jacobs, Miia Kivipelto, Rainer Rauramaa

**Affiliations:** Kuopio Research Institute of Exercise Medicine, Kuopio, Finland; Department of Chronic Disease Prevention, National Institute of Health and Welfare, Helsinki, Finland; Dasman Diabetes Institute, Dasman, Kuwait; Kuopio Research Institute of Exercise Medicine, Kuopio, Finland; Department of Clinical Physiology and Nuclear Medicine, Kuopio University Hospital, Kuopio, Finland; Kuopio Research Institute of Exercise Medicine, Kuopio, Finland; Institute of Public Health and Clinical Nutrition, University of Eastern Finland, Kuopio, Finland; Kuopio Research Institute of Exercise Medicine, Kuopio, Finland; Kuopio Research Institute of Exercise Medicine, Kuopio, Finland; Department of Clinical Physiology and Nuclear Medicine, Kuopio University Hospital, Kuopio, Finland; Institute of Biomedicine/Physiology, University of Eastern Finland, Kuopio Campus, Kuopio, Finland; Department of Neurology, Kuopio University Hospital, Kuopio, Finland; Finnish Medicines Agency, Kuopio, Finland; Division of Epidemiology and Community Health, School of Public Health, University of Minnesota, Minnesota, MN, USA; Department of Neuroscience and Neurology, University of Eastern Finland, Kuopio Campus, Kuopio, Finland; Department of Neurobiology, Care Sciences, and Society, Division of Clinical Geriatrics, Karolinska Institutet and Karolinska University Hospital, Stockholm, Sweden; Kuopio Research Institute of Exercise Medicine, Kuopio, Finland

**Keywords:** aerobic exercise, resistance exercise, healthy diet, cognitive function, older individuals

## Abstract

**Background:**

Evidence for the effects of exercise and dietary interventions on cognition from long-term randomized controlled trials (RCTs) in large general populations remains insufficient.

**Objective:**

The objective of our study was to investigate the independent and combined effects of resistance and aerobic exercise and dietary interventions on cognition in a population sample of middle-aged and older individuals.

**Methods:**

We conducted a 4-y RCT in 1401 men and women aged 57–78 y at baseline. The participants were randomly assigned to the resistance exercise, aerobic exercise, diet, combined resistance exercise and diet, combined aerobic exercise and diet, or control group. Exercise goals were at least moderate-intensity resistance exercise ≥2 times/wk and at least moderate-intensity aerobic exercise ≥5 times/wk. Dietary goals were ≥400 g/d of vegetables, fruit, and berries; ≥2 servings of fish/wk; ≥14 g fiber/1000 kcal; and ≤10% of energy of daily energy intake from SFAs. The primary outcome was the change in global cognition measured by the total score of the Consortium to Establish a Registry for Alzheimer's Disease (CERAD) neuropsychological tests [CERAD total score (CERAD-TS)]. The data were analyzed using the intention-to-treat principle and linear mixed-effects models.

**Results:**

There was a trend toward improved CERAD-TS over 4 y in the combined aerobic exercise and diet group compared with the control group (net increase: 1.4 points; 95% CI: 0.1, 2.7; *P* = 0.06) adjusted for age, sex, years of education, symptoms of depression, and waist circumference at baseline. No other differences in CERAD-TS changes were found across the 6 study groups. Diet did not potentiate the effect of aerobic or resistance exercise on CERAD-TS.

**Conclusions:**

A combination of at least moderate-intensity aerobic exercise and a healthy diet may improve cognition in older individuals over 4 y, but there was no effect of either of these interventions alone, resistance training alone, or resistance exercise with a healthy diet on cognition.

See corresponding editorial on page 1392.

## Introduction

Previous studies suggest that aerobic and resistance exercise mitigate age-related cognitive impairment (1–3). However, such evidence is mainly based on randomized controlled trials (RCTs) with relatively small numbers of participants, short follow-up periods, and inconsistent results (4–7). Therefore, it has been emphasized that more research is needed on the effects of aerobic and resistance exercise on cognitive function in older people (4–7).

There is some evidence for the preventive effect of a healthy diet on cognitive decline with aging, but it mainly comes from prospective epidemiological studies (8–12). RCTs in older people at increased risk of cardiovascular disease have found beneficial effects of a healthy diet on cognition (13, 14), whereas no such effect was observed in a relatively short-term RCT in cognitively healthy older individuals (15).

There are few intervention studies on the combined effects of physical exercise and a healthy diet on cognition, particularly in general populations (16). One RCT showed that aerobic and resistance exercise, including flexibility and balance training, combined with a calorie-controlled diet improved cognition in older cognitively healthy individuals with obesity, sedentariness, and frailty (3). Another RCT showed that a multicomponent intervention, including physical exercise, a healthy diet, cognitive training, stimulating social activity, and cardiovascular risk monitoring, prevented cognitive decline in middle-aged and older individuals at increased risk of dementia (17).

A common conclusion of systematic reviews and meta-analyses is the need for long-term RCTs on the effects of aerobic and resistance exercise and a healthy diet on cognitive function in large study samples (5–10, 18). We therefore carried out a 4-y RCT to investigate whether resistance or aerobic exercise or a healthy diet alone or their combinations decrease age-related cognitive decline in a general population of middle-aged and older men and women. We tested a predefined hypothesis that resistance exercise, aerobic exercise, and a healthy diet alone and combinations of resistance or aerobic exercise and a healthy diet would decrease cognitive decline with aging compared with no intervention. We also hypothesized that combinations of resistance or aerobic exercise and a healthy diet are more effective than aerobic or resistance exercise or a healthy diet alone.

## Methods

### Study design and participants

The Dose-Responses to Exercise Training (DR's EXTRA) study is a 4-y RCT on the health effects of regular physical exercise and a healthy diet in a population-based random sample of Finnish men and women aged 55–74 y living in the city of Kuopio in 2002 (see **Supplemental Figure 1**). The 3000 men and women who were invited to participate in the study were obtained from the national population registry, and finally 1479 of them attended the baseline measurements between 5 April in 2005 and 4 October in 2006. The prespecified exclusion criteria were medical or other conditions that prohibit engagement in an exercise intervention or the assessments, as judged by a physician (see more details in **Supplemental Table 1**). After these exclusions, 1410 individuals aged 57–78 y at baseline in 2005–2006 were randomly assigned to the resistance exercise, aerobic exercise, diet, combined resistance exercise and diet, combined aerobic exercise and diet, or control group. After removing 2 individuals with missing data on the Consortium to Establish a Registry for Alzheimer's Disease (CERAD) total score (CERAD-TS) and 7 individuals who were not native speakers of Finnish and thereby unable to fill out the questionnaires in Finnish, there were 1401 individuals in the present analyses. Couples (*n* = 41) were included in the trial but randomly assigned individually. Two-year measurements were performed between 22 May in 2007 and 17 December in 2008, and 4-y measurements were performed between 5 October in 2009 and 15 March in 2011. The study complies with the Declaration of Helsinki, and the protocol was approved by the Research Ethics Committee of the Hospital District of Northern Savo, Finland. The participants gave signed informed consent.

### Randomization and blinding

The participants were randomly assigned in a balanced fashion to 6 study groups in blocks of 180 participants with the order of the group assignments within each block being random under surveillance of the principal investigator (**Table 1**). This procedure involved the participants choosing one of the identically sealed opaque envelopes in sequential order that contained the group assignment. The principal investigator did not participate in baseline measurements and was blinded for the outcome measures. The investigators who performed or evaluated the outcome measures were blinded to the study groups.

### Interventions

In the aerobic exercise group, the resistance exercise group, and the diet group, the participants had a total of 11 individualized face-to-face counseling sessions of 30 min carried out by 5 exercise physiologists and 2 authorized nutritionists over 4 y. In the combined aerobic exercise and diet group and in the combined resistance exercise and diet group, the intervention thus included up to 22 individualized face-to-face counseling sessions. The first 5 of the counseling sessions occurred during the first year and the other 6 sessions took place every sixth month during the last 3 y. The exercise physiologists and the nutritionists had a predefined topic according to which they followed the intervention prescriptions. The main purpose of all face-to-face counseling sessions was to monitor realization of the interventions as planned and motivate participants for the long intervention. During individual counseling sessions the participants were also queried about possible adverse events related to the interventions.

To further improve motivation and adoption to the interventions, we also provided group counseling sessions in groups of 15–20 participants for all intervention groups. In the aerobic exercise group, the resistance exercise group, and the diet group, the participants had 3 group counseling sessions carried out by the exercise physiologists and the nutritionists. The first counseling session was carried out during the first 3 mo, the second between 6 and 12 mo, and the third after 24 mo from the beginning of the study. In the combined aerobic exercise and diet group and in the combined resistance exercise and diet group, the intervention thus included 6 group counseling sessions. The main purpose of the group counseling sessions was to offer practical advice for the participants to improve their health behavior and achieve gradual, permanent lifestyle changes in diet and/or physical exercise. For example, the participants in the diet groups were advised on how to read and understand package markings to identify the proper food products in the shops, the participants in the aerobic exercise groups were advised about the correct Nordic walking technique, and the participants in the resistance exercise groups received explanation of the importance of muscle strength for functional capacity. The spouses of the participants could also attend all group counseling sessions. However, they could attend the counseling sessions together only if their sessions were scheduled on the same date/time and if they were randomly assigned to the same treatment group.

In resistance exercise, training load was quantified based on 1 repetition maximum (RM) assessed by 3–5 RM or 16–20 RM tests (19) for main muscle groups (i.e., knee extension and flexion, abdomen and back muscles, rotation, upper back and arm muscles, and press bench for lower extremity muscles). The training loads were adjusted on demand throughout the 4-y intervention period and RM tests were carried out at 1, 3, 6, 24, 36, and 48 mo. The training load in the resistance exercise group was started with 1 strength-training session/wk, 1 set for main muscle groups (knee extension and flexion, abdomen and back muscles, rotation, upper back and arm muscles, and press bench for lower extremity muscles) and 10 repetitions for each set at a load of 40% of estimated 1 RM for the first 6 mo. Thereafter, the purpose was to continue resistance exercise for at least 2 strength-training sessions/wk, 2 sets for main muscle groups per each session, and 15 repetitions for each muscle group in a set at a load of 60% of estimated 1 RM. Resistance exercise was conducted in the gym in the research center and guided by the exercise physiologist. For resistance exercise, air resistance computer-based equipment with a smart card system (HUR Ltd.) was used. Exercise prescription was loaded on a smart card and training data were stored via the card to the computer.

The participants in the aerobic exercise group were prescribed an individualized progressive intervention program by an exercise physiologist. Training frequency, duration, and intensity were gradually increased from 2 to 4 times/wk at an intensity corresponding to ∼40% to 50% of maximal oxygen uptake measured individually in a maximal exercise test and lasting 30 to 60 min/session during the first 6 mo. Thereafter, the purpose was to continue at least 60 min of aerobic exercise 5 times/wk at an intensity corresponding to ∼60% of maximal oxygen uptake measured individually in a maximal exercise test. The participants in the aerobic exercise group performed exercise on their own—that is, they performed training by themselves, without supervision, and were instructed to monitor training intensity via a heart rate monitor or by palpating arterial pulse. The personal characteristics of participants, such as preferred forms of aerobic exercise, overall health, and possibilities to carry out the program, were taken into account in the aerobic exercise intervention planning.

The participants in the diet group were instructed to follow the Finnish Nutrition Recommendations, which were in line with nutrition recommendations for diabetes (20) and included ≥400 g/d of vegetables, fruit, and berries; ≥2 servings of fish/week corresponding to ≥30 g/d; ≥14 g of fiber/1000 kcal; and ≤10% of energy (E%) of daily energy intake from SFAs. The dietary instructions were tailored based on the usual diet and current health status of participants. All dietary instructions were given at a food level—for example, instead of an abstract goal to decrease intake of saturated fat, the instruction at a food level was to substitute high-fat dairy products with low-fat dairy products. Spouses, if they were in charge of preparing meals at home, were asked to be present at the advice sessions.

The combined resistance or aerobic exercise and diet groups had similar purposes and followed the intervention prescriptions explained above for a single group. Due to ethical reasons, the participants in the control group were reminded about the general recommendations on physical activity and diet at baseline.

### Assessment of cognitive function

Cognitive function was assessed at baseline and at 2-y and 4-y follow-ups using the standardized Finnish translation of the CERAD neuropsychological tests, including the Mini-Mental State Examination (MMSE) (21). Three nurses who performed the CERAD tests were trained by a neuropsychologist. We calculated CERAD-TS to measure global cognitive performance by summing the components of CERAD-TS, including Verbal Fluency, Modified Boston Naming, Word List Memory, Constructional Praxis, Word List Recall, and Word List Recognition (22). The maximum of CERAD-TS was 100 points, with a higher score indicating better performance.

### Assessment of physical activity

At least moderate-intensity physical activity from the previous 12 mo was assessed using a 12-mo leisure-time physical activity questionnaire (23) that was modified from the Minnesota Leisure Time Physical Activity Questionnaire (24). The questionnaire included the most common leisure-time physical activities of Finnish men and women, such as walking, Nordic walking, cycling, commuting walking, commuting cycling, jogging, running, orienteering, cross-country skiing, skating, rowing, paddling, swimming, water gymnastics, golf, other ball games, downhill skiing and snowboarding, dancing, bowling, aerobics and group and home-based gymnastic exercises, and resistance training. The participants filled out the frequency of each physical activity per month during the previous 12 mo, the mean duration of a single session, and the mean intensity of each physical activity scored as 1 = light, 2 = moderate, 3 = heavy, and 4 = very heavy. The exercise physiologist or a trained nurse completed the form, if needed. The frequency of each physical activity per month, the duration of each session, and the intensity of physical activity were multiplied to calculate metabolic equivalent (MET)-hours per week. The MET values for each intensity level of physical activity scored 1–4 were determined based on the means of maximal METs achieved during the maximal exercise tests in 22 age- and sex-specific groups (25). One MET refers to the resting metabolic rate and corresponds to the oxygen consumption of 3.5 mL · kg^−1^/min^−1^.

### Assessment of diet

Dietary intake was assessed by a 4-d food record of predefined consecutive days, including 3 weekdays and 1 weekend day (26). The participants received the food records with detailed verbal and written instructions at the first study visit and returned them at the second visit 1 wk later. The amount of food consumed was estimated by a picture booklet of portion sizes (27) using household gauges or weighing. The food records were completed by a clinical nutritionist or a trained nurse, if needed. Data from food records were analyzed using the MicroNutrica^®^ nutrient calculation software, version 2.5 (Finnish Social Insurance Institution, version 2.5). The recipes for foodstuff to respond to selection of foodstuff in shops, to change nutritional content to respond to existing situation of margarines, as well as to add some recipes of single dishes were updated by the clinical nutritionists in 2007. Potential over- or underreporting in dietary data was not formally analyzed.

### Other assessments

Body weight was measured with a digital scale and body height using a metal-scaled height meter. BMI was calculated by dividing weight by height squared. Obesity was defined as BMI (kg/m^2^) ≥30. Waist circumference was measured twice on bare skin at mid-distance between the bottom of the rib cage and the top of the iliac crest, and the mean of 2 measurements was used. Blood samples were taken after a 12-h fast. Serum total, LDL, and HDL cholesterol and triglycerides were measured by enzymatic photometric methods. Fasting plasma glucose was measured by the hexokinase method. Blood pressure was recorded from the right arm in a sitting position after a 5-min rest using a mercury sphygmomanometer. Two independent consecutive measurements of systolic and diastolic blood pressure were taken, and the mean of the measurements was used in the analyses. We used maximal oxygen uptake as a measure of cardiorespiratory fitness and assessed it in a maximal symptom-limited exercise test on a cycle ergometer. Prevalent diseases diagnosed by a physician, the use of medications, education, alcohol consumption, and smoking were assessed by self-administered questionnaires. The symptoms of depression were assessed by the Center for Epidemiologic Studies–Depression Scale (28).

### Power calculation

In the DR's EXTRA study we have 3 prespecified primary outcomes: the 4-y change in carotid artery intima-media thickness (IMT), endothelial function, and cognitive function. The power calculations were based on the 4-y increase in carotid IMT in the DNA Polymorphism and Carotid Atherosclerosis (DNASCO) Study (29) because we assumed that it requires a larger number of participants than the other 2 main outcomes. Thus, the prespecified power calculation with CERAD-TS as an outcome was not performed. Retrospectively, given the variance observed in baseline CERAD-TS values, the sample size in control, aerobic exercise, resistance exercise, and diet groups in the current study provided 98% power to detect the difference of 3.5 points (a minimal clinically meaningful difference) in CERAD-TS between any 2 groups with a 5% 2-sided ɑ. As combined groups of aerobic or resistance exercise and a healthy diet were supposed to bring about an even larger effect than single interventions compared with the control group, the sample sizes of the combination groups were assumed to be more than adequate to detect the target difference of 3 points in CERAD-TS.

### Calculation of compliance

According to our definitions below, compliance ranged between 0% and 100%, depending on how the participant followed the intervention prescription. The participants in the aerobic exercise group were defined to be 100% compliant to the intervention if they had at least 300 min (60 min/session × 5 sessions) of moderate-intensity aerobic exercise/wk. For example, compliance of 60% means that the participant reported 180 min of aerobic exercise/wk. The data for compliance to the aerobic exercise were obtained from a 12-mo leisure-time physical activity questionnaire. The participants in the resistance exercise group were defined as 100% compliant to the intervention if they had at least 2 strength-training sessions/wk, 2 sets for main muscle groups (i.e., knee extension and flexion, abdomen and back muscles, rotation, upper back and arm muscles, and press bench for lower extremity muscles per session), and 15 repetitions for each set at a load of 60% of estimated 1 RM (19). The data for compliance to the resistance exercise were obtained from a computerized training system that utilized a smart card to store training data (HUR Ltd.). The participants in the diet group were defined as 100% compliant to each of the components of the intervention if they consumed ≥400 g/d of vegetables, fruit, and berries; ≥2 servings of fish/wk corresponding to ≥30 g/d; ≥14 g fiber/1000 kcal; and ≤10 E% of daily energy intake from SFAs (20). They were defined as 100% compliant to the whole dietary intervention if compliance for all components was 100%. Compliance to the combined resistance or aerobic exercise and dietary intervention was the mean compliance to these interventions. Spousal support in compliance and possibly in the magnitude of the treatment effect was not investigated.

### Statistical analyses

The main outcome in the present analyses was the 4-y change in CERAD-TS (21). Continuous variables are shown as means and SDs and dichotomous variables as frequencies and percentages. Data were analyzed according to intention-to-treat (ITT) by using a linear mixed model according to a 2-level structure—that is, repeated CERAD-TS measures (baseline, 2 y, 4 y) were clustered within the subjects. To compare models with different variance-covariance structures, the Bayesian information criterion (BIC) was used to find the optimal model. BIC is an indicator of model fit, based on the −2 log likelihood, but taking the number of parameters estimated into account.

In the primary analysis we analyzed the data as a 2 × 3 factorial design in which a dichotomous indicator of treatment was coded separately for aerobic exercise, resistance exercise, and diet, and time was coded as a continuous variable. The indicators of treatment were deliberately omitted from the model to allow for a regression-to-the-mean phenomenon resulting from a random baseline imbalance in CERAD-TS values (30). This approach provides an elegant way to adjust for the differences in CERAD-TS at baseline without inclusion of the baseline value itself in the model. The covariates age, gender, years of education, symptoms of depression at baseline, and waist circumference at baseline were forced in the adjusted model. The current data showed the best fit with the model in which both a random intercept and a random effect for regression coefficient of time were modeled by using a scaled identity variance structure, as follows:

(1) \begin{eqnarray*} &&{\rm{CERAD - T}}{{\rm{S}}_{{\rm{it}}}}\nonumber\\ &&\quad = {\beta _0}+{\beta _1}\,\,{\rm{(age)}}+{\beta _2}\,\,{\rm{(gender)}}+{\beta _3}\,\,{\rm{(education}}\,\,{\rm{years)}}\nonumber\\ &&\qquad +\, {\beta _4}\,\,{\rm{(symptoms}}\,\,{\rm{of}}\,\,{\rm{depression}}\,\,{\rm{at}}\,\,{\rm{baseline) }}\nonumber\\ &&\qquad +\, {\beta _5}\,\,{\rm{(waist}}\,\,{\rm{circumference}}\,\,{\rm{at}}\,\,{\rm{baseline)}} + {\beta _6}\,\,{\rm{(time)}}\nonumber\\ &&\qquad +\, {\beta _7}\,\,{\rm{(aerobic}}\,\,{\rm{exercise}} \times {\rm{time) }} + {\beta _8}\,\,{\rm{(resistance}}\,\,{\rm{exercise}}\nonumber\\ &&\qquad \times\, {\rm{time)}} + {\beta _9}\,\,{\rm{(diet}} \times {\rm{time)}} + {\beta _{10}}\,\,{\rm{(aerobic}}\,\,{\rm{exercise}} \times {\rm{diet}}\nonumber\\ &&\qquad \times\, {\rm{time) }} + {\beta _{11}}\,\,{\rm{(resistance}}\,\,{\rm{exercise}} \times {\rm{diet}} \times {\rm{time)}} + {u_{0i}}\nonumber\\ &&\qquad +\, {u_i}\,\,{\rm{(tim}}{{\rm{e)}} }+{\varepsilon _{{\rm{it}}}} \end{eqnarray*}

where β_0_ is the intercept; β_1_, β_2_, β_3_, β_4_, and β_5_ reflect the independent effects of each covariate (age, gender, education years, symptoms of depression, waist circumference at baseline); β_6_ reflects the covariate-adjusted change in CERAD-TS in the control group over the 4-y intervention period; β_7_, β_8_, and β_9_ reflect the individual adjusted effects of aerobic exercise, resistance exercise, and diet compared with control group, respectively; β_10_ reflects the adjusted effect of the combination of diet with aerobic exercise compared with the sum of their individual effects; and β_11_ reflects the adjusted effect of the combination of diet with resistance exercise compared with the sum of their individual effects.

From the model described above, the adjusted contrasts between groups of aerobic or resistance exercise combined with a healthy diet and the control group cannot be directly derived. To enable that comparison, another model was built in which 3 dichotomous indicators of treatment (used in the first model) were replaced by 5 actual intervention groups.

The nonlinear change across time was explored by adding a quadratic term to the model and by logarithmic transformation, but this did not improve the fit of the linear model. To examine the intervention effects during the first 2 intervention years, another model was built with time as a categorical variable (baseline, 2 y, 4 y).

The difference in the total 4-y drop-out rate between the study groups was analyzed using the chi-square test. Hedges’ *g* as a measure of effect size (ES) for the estimated difference in 4-y change in CERAD-TS between any intervention group and the control group was calculated by using a given estimated contrast derived from the linear mixed model as a nominator and a pooled SD at baseline as a denominator. The 95% CIs for ES were derived from noncentral *t* distribution (31).

The validity of the assumption of normality of the residuals was verified by inspection of a quantile-quantile (or a normal probability) plot. Plotting residuals against fitted values was used for an assessment of linearity and homoscedasticity assumptions. No violations were observed. Various covariance structures were explored to adjust for the correlated observations within the subject after including a random intercept and a random effect for the regression coefficient of time in a final model (see above). A variance component structure assuming a single constant variance for measurement occasions but no covariances between occasions was found to optimize the model based on the BIC value. Autocorrelation was assessed by calculating the variance inflation factor (VIF) for each variable of interest (aerobic exercise, resistance exercise, diet). Not unexpectedly, considering the RCT design, VIFs ranged from 1.01 to 1.69, indicating negligible autocorrelation.

Because all of the missing data were assumed to be missing at random, no method was applied to impute missing values. *P* values observed in analyses for each outcome were corrected for multiple comparisons using the 2-stage Benjamini-Hochberg procedure (32), which controls for the false discovery rate regardless of ES or degree of dependence between tests (33). Within the 2-stage correction approach described, the comparisons between dropouts and those who completed the trial were considered as 1 cluster of tests, whereas all other *P* values derived from the trial design itself were considered as a separate cluster of tests.

All statistical analyses were 2-sided, and adjusted *P* values of <0.05 were considered statistically significant. The IBM SPSS Statistics for Windows, version 24.0 (IBM Corporation), was used for all analyses.

## Results

### Study characteristics

Baseline characteristics showed a balanced randomization across the study groups (Table 1). The mean ± SD age, education duration, CERAD-TS, and MMSE were 66.5 ± 5.4 y, 11.2 ± 3.9 y, 82.5 ± 9.2 points, and 27.6 ± 2.0 points, respectively. At baseline, 54% of all participants reached the current recommendation for at least moderate-intensity aerobic exercise (≥150 min/wk), whereas <1% reported no resistance exercise. Altogether, 40% of all participants achieved the current recommendation for the consumption of vegetables, fruit, and berries (≥400 g/d); 54% for the consumption of fish (≥2 servings/wk); 44% for the intake of fiber (≥14 g/1000 kcal); and 33% for the intake of saturated fat (≤10 E%).

**TABLE 1:** Baseline characteristics of the participants in the 6 study groups^1^

	Control (*n* = 234)	Resistance exercise (*n* = 234)	Aerobic exercise (*n* = 234)	Diet (*n* = 235)	Resistance exercise + diet (*n* = 232)	Aerobic exercise + diet (*n* = 232)
Men, *n* (%)	123 (53)	119 (51)	111 (47)	121 (52)	107 (46)	110 (46)
Age, y	66.2 ± 5.4	66.6 ± 5.4	67.0 ± 5.3	67.0 ± 5.5	65.8 ± 5.4	66.2 ± 5.3
Education, y	11.2 ± 3.9	11.3 ± 3.9	11.4 ± 4.0	11.2 ± 3.6	10.7 ± 3.6	11.4 ± 4.1
Mini-Mental State Examination, points	27.5 ± 2.2	27.6 ± 1.9	27.5 ± 1.9	27.7 ± 1.8	27.5 ± 2.2	27.5 ± 1.8
At least moderate-intensity physical exercise, min/wk	187 ± 181	202 ± 197	189 ± 181	199 ± 197	207 ± 209	197 ± 197
Total energy intake, kcal/d	1685 ± 461	1670 ± 460	1705 ± 474	1684 ± 454	1647 ± 445	1663 ± 441
Energy intake from SFAs, E%/d	11.9 ± 3.3	11.8 ± 3.3	11.5 ± 2.9	11.3 ± 2.9	11.2 ± 2.8	11.1 ± 2.9
Maximal oxygen uptake, L/min	1.9 ± 0.6	1.8 ± 0.6	1.8 ± 0.5	1.8 ± 0.6	1.8 ± 0.5	1.8 ± 0.5
Alcohol consumption, doses/previous week	4.6 ± 6.4	4.2 ± 6.8	4.3 ± 6.2	4.3 ± 6.6	3.4 ± 6.0	4.7 ± 8.5
Smoking, never/past/current, *n* (%)	108/100/26 (46/43/11)	136/68/30 (58/29/13)	137/77/20 (59/33/9)	135/76/24 (57/32/10)	118/87/27 (51/38/12)	132/77/23 (57/33/10)
Waist circumference, cm	94.4 ± 12.8	93.0 ± 12.6	94.8 ± 12.7	93.2 ± 12.5	92.8 ± 13.7	94.7 ± 14.0
BMI, kg/m^2^	27.7 ± 4.4	27.3 ± 4.3	28.2 ± 4.4	27.4 ± 4.5	27.4 ± 4.6	28.0 ± 4.8
Serum total cholesterol, mmol/L	5.1 ± 0.9	5.1 ± 1.0	5.0 ± 1.0	5.1 ± 0.9	5.0 ± 0.9	5.1 ± 0.9
Serum LDL cholesterol, mmol/L	3.2 ± 0.8	3.2 ± 0.9	3.1 ± 0.9	3.3 ± 0.8	3.2 ± 0.9	3.2 ± 0.8
Serum HDL cholesterol, mmol/L	1.7 ± 0.5	1.7 ± 0.5	1.7 ± 0.5	1.7 ± 0.5	1.7 ± 0.5	1.7 ± 0.5
Serum triglycerides, mmol/L	1.3 ± 0.7	1.3 ± 0.7	1.3 ± 0.7	1.4 ± 0.7	1.4 ± 0.7	1.3 ± 0.8
Plasma glucose, mmol/L	5.8 ± 0.8	5.8 ± 0.9	5.9 ± 1.1	5.8 ± 0.9	5.9 ± 1.2	5.8 ± 0.9
Systolic blood pressure, mm Hg	147.2 ± 20.2	147.2 ± 20.8	149.5 ± 20.3	148.7 ± 19.1	147.2 ± 21.0	146.8 ± 19.6
Diastolic blood pressure, mm Hg	83.0 ± 9.4	82.6 ± 9.4	83.8 ± 8.8	83.5 ± 9.6	83.5 ± 8.9	83.5 ± 9.7
Obesity, *n* (%)	56 (24)	53 (23)	67 (29)	48 (20)	54 (23)	65 (28)
Metabolic syndrome, *n* (%)	63 (27)	54 (23)	65 (28)	57 (24)	60 (26)	68 (29)
Type 2 diabetes, *n* (%)	17 (7)	14 (6)	21 (9)	23 (10)	20 (9)	21 (9)
Hypertension, *n* (%)	100 (43)	105 (45)	122 (52)	98 (42)	112 (48)	113 (49)
Coronary artery disease, *n* (%)	33 (14)	32 (14)	42 (18)	30 (13)	33 (14)	35 (15)
Cardiac insufficiency, *n* (%)	10 (4)	8 (3)	6 (3)	8 (3)	11 (5)	9 (4)
Lower extremity peripheral artery disease, *n* (%)	7 (3)	6 (3)	5 (2)	7 (3)	7 (3)	10 (4)
History of stroke, *n* (%)	10 (4)	11 (5)	8 (3)	4 (2)	16 (7)	8 (3)
History of transient ischemic attack, *n* (%)	13 (6)	17 (7)	17 (7)	11 (5)	16 (7)	12 (5)
History of cancer, *n* (%)	22 (9)	28 (12)	20 (9)	24 (10)	20 (9)	22 (10)
Pulmonary disease, *n* (%)	61 (26)	58 (25)	54 (23)	46 (20)	49 (21)	48 (21)
Joint disease, *n* (%)	105 (45)	101 (43)	120 (51)	108 (46)	106 (46)	103 (44)
Symptoms of depression, *n* (%)	27 (12)	30 (13)	34 (15)	25 (11)	31 (13)	38 (16)
Lipid-lowering medication, *n* (%)	79 (34)	74 (32)	92 (39)	80 (34)	88 (38)	76 (33)
Antihypertensive medication, *n* (%)	93 (40)	99 (42)	112 (48)	90 (38)	99 (43)	96 (41)
Glucose-lowering medication, *n* (%)	15 (6)	14 (6)	18 (8)	19 (8)	16 (6)	18 (8)

^1^Values are means ± SDs or *n* (%). Coronary artery disease includes angina pectoris, myocardial infarction, coronary artery bypass surgery, and percutaneous transluminal coronary artery angioplasty; stroke includes ischemic and hemorrhagic stroke; 33 cL of regular beer or other corresponding alcohol drink, ∼4.7 vol% alcohol; 12 cL of normal wine, ∼12 vol% alcohol; 8 cL of strong wine, ∼19 vol% alcohol; and 4 cL of distilled spirits, ∼40 vol% alcohol are considered 1 dose = 12 g alcohol. E%, percentage of energy.

The median (IQR) follow-up period was 4.4 (4.2, 4.5) y. Altogether, 211 (15%) of the participants dropped out during the 4-y follow-up with no difference between the study groups (*P* = 0.50) (see Supplemental Figure 1). Those who dropped out were older (68.2 ± 5.8 y vs. 66.1 ± 5.2 y, *P* = 0.003), had a lower CERAD-TS (79.0 ± 0.7 points vs. 83.1 ± 8.8 points, *P* = 0.003), a higher BMI (28.3 ± 5.4 vs. 27.5 ± 4.3, *P* = 0.02), and consumed less vegetables, fruit, and berries (352.1 ± 208.2 g/d vs. 393.0 ± 198.5 g/d, *P* = 0.01) and fish (37.6 ± 47.6 g/d vs. 47.6 ± 50.3 g/d, *P* = 0.01), but had similar amounts of at least moderate-intensity aerobic exercise (240.7 ± 274.2 min/wk vs. 247.5 ± 259.0 min/wk, *P* = 0.73) at baseline compared with the 1199 participants who completed the trial.

### Changes in physical exercise and diet during 4 y

The frequency (times/wk) of resistance exercise increased in the resistance exercise group and in the combined resistance exercise and diet group. Similarly, duration (min/wk) and volume (MET-h/wk) of aerobic exercise increased in the aerobic exercise group and slightly increased in the combined aerobic exercise and diet group (**Table 2**). Participants in the diet group, in the combined resistance exercise and diet group, and in the combined aerobic exercise and diet group reached, on average, at least 3 out of 4 dietary goals. Participants in the control group maintained or slightly increased physical exercise and maintained or improved diet quality. The mean compliance to the prescribed interventions was 53% in the resistance exercise group, 62% in the aerobic exercise group, 84% in the diet group, 66% in the combined resistance exercise and diet group (47% for resistance exercise and 84% for diet), and 71% in the combined aerobic exercise and diet group (57% for aerobic exercise and 85% for diet). No relevant intervention-related adverse events were reported.

**TABLE 2:** Estimated volume, duration, and frequency of at least moderate-intensity aerobic exercise, frequency of resistance exercise, and dietary goals at baseline as well as their estimated changes during 4 y^1^

Intervention goal	Control (*n* = 234)	Resistance (*n* = 234)	Aerobic (*n* = 234)	Diet (*n* = 235)	Resistance exercise + diet (*n* = 232)	Aerobic exercise + diet (*n* = 232)
Aerobic exercise						
Volume, MET-h/wk						
Baseline	12.0 (10.4, 13.7)	13.1 (11.5, 14.8)	12.1 (10.4, 13.7)	12.7 (11.1, 14.3)	13.1 (11.5, 14.8)	12.8 (11.1, 14.4)
Baseline to 2 y	0.1 (−1.9, 2.0)	0.2 (−1.8, 2.2)	4.3 (2.3, 6.2)	−0.1 (−2.1, 1.9)	−1.6 (−3.6, 0.4)	1.8 (−0.2, 3.8)
Baseline to 4 y	−0.4 (−2.5, 1.6)	−2.1 (−4.1, −0.02)	2.7 (0.7, 4.7)	−0.6 (−2.7, 1.4)	−2.2 (−4.3, −0.2)	1.7 (−0.3, 3.8)
*P* for 4-y change within group	0.82	0.05	0.001	0.75	0.10	0.06
*P* for difference in 4-y change between the intervention group and the control group		0.25	0.04	0.88	0.20	0.13
Duration, min/wk						
Baseline	182.8 (157.7, 207.9)	202.9 (177.9, 228.0)	189.1 (164.0, 214.2)	199.3 (174.3, 224.4)	205.4 (180.1, 230.7)	197.2 (171.9, 222.4)
Baseline to 2 y	5.1 (−25.6, 35.9)	3.1 (−28.1, 34.3)	66.6 (35.9, 97.3)	−0.1 (−31.1, 30.9)	−23.7 (−55.0, 7.6)	26.6 (−4.3, 57.5)
Baseline to 4 y	2.8 (−29.0, 34.5)	−21.4 (−53.1, 10.3)	47.1 (15.6, 78.7)	−2.3 (−34.4, 29.7)	−29.7 (−62.1, 2.7)	31.2 (−0.2, 62.6)
*P* for 4-y change within group	0.92	0.20	0.001	0.98	0.11	0.06
*P* for difference in 4-y change between the intervention group and the control group		0.28	0.04	0.78	0.14	0.20
Resistance exercise						
Frequency, times/wk						
Baseline	0.1 (−0.004, 0.2)	0.1 (0.1, 0.2)	0.1 (0.02, 0.2)	0.1 (0.1, 0.2)	0.2 (0.1, 0.3)	0.1 (−0.003, 0.2)
Baseline to 2 y	0.1 (−0.03, 0.2)	1.5 (1.4, 1.6)	0.05 (−0.07, 0.2)	0.1 (−0.04, 0.2)	1.2 (1.1, 1.3)	0.01 (−0.1, 0.1)
Baseline to 4 y	0.1 (0.02, 0.3)	1.3 (1.2, 1.4)	0.2 (0.04, 0.3)	0.1 (0.003, 0.2)	1.1 (1.0, 1.2)	0.1 (−0.01, 0.2)
*P* for 4-y change within group	0.05	0.001	0.03	0.11	0.001	0.05
*P* for difference in 4-y change between the intervention group and the control group		0.001	0.73	0.87	0.001	0.72
Diet						
Vegetables, fruit, berries, g/d						
Baseline	375.4 (348.9, 401.9)	385.7 (359.1, 412.2)	399.4 (373.0, 425.9)	412.0 (385.5, 438.4)	389.1 (362.7, 415.6)	396.2 (369.6, 422.8)
Baseline to 2 y	8.3 (−21.4, 38.0)	21.7 (−8.5, 52.0)	1.5 (−28.1, 31.1)	54.4 (24.6, 84.3)	58.6 (28.6, 88.5)	38.0 (8.2, 67.8)
Baseline to 4 y	34.0 (3.4, 64.5)	6.8 (−23.9, 37.5)	−13.9 (−44.2, 16.4)	29.5 (−1.4, 60.3)	58.0 (27.0, 89.0)	31.7 (1.5, 62.0)
*P* for 4-y change within group	0.05	0.27	0.43	0.001	0.001	0.03
*P* for difference in 4-y change between the intervention group and the control group		0.21	0.15	0.93	0.23	0.93
Dietary fiber, g/1000 kcal						
Baseline	13.5 (13.0, 14.0)	13.7 (13.2, 14.2)	13.6 (13.1, 14.1)	13.7 (13.2, 14.2)	14.0 (13.5, 14.5)	14.0 (13.5, 14.5)
Baseline to 2 y	−0.1 (−0.7, 0.5)	0.4 (−0.2, 1.0)	0.6 (0.02, 1.2)	1.1 (0.5, 1.7)	0.7 (0.1, 1.3)	0.8 (0.2, 1.4)
Baseline to 4 y	−0.4 (−1.0, 0.2)	−0.2 (−0.8, 0.4)	−0.4 (−1.0, 0.3)	0.6 (−0.04, 1.2)	0.3 (−0.3, 1.0)	0.2 (−0.4, 0.8)
*P* for 4-y change within group	0.32	0.06	0.01	0.001	0.04	0.03
*P* for difference in 4-y change between the intervention group and the control group		0.65	0.86	0.03	0.08	0.15
Fish, g/d						
Baseline	45.6 (39.0, 52.2)	50.6 (44.0, 57.2)	44.8 (38.2, 51.4)	44.7 (38.1, 51.3)	46.6 (40.0, 53.2)	43.8 (37.2, 50.5)
Baseline to 2 y	1.1 (−9.0, 11.2)	−5.02 (−15.4, 5.0)	11.1 (1.1, 21.2)	1.7 (−8.4, 11.8)	4.9 (−5.2, 15.1)	7.6 (−2.5, 17.7)
Baseline to 4 y	6.7 (−3.6, 17.0)	1.0 (−9.4, 11.3)	5.5 (−4.8, 15.8)	7.5 (−2.9, 17.9)	5.9 (−4.6, 16.4)	4.4 (−5.8, 14.7)
*P* for 4-y change within group	0.31	0.35	0.06	0.25	0.38	0.25
*P* for difference in 4-y change between the intervention group and the control group		0.38	0.91	0.90	0.93	0.77
Saturated fat, E%						
Baseline	11.9 (11.5, 12.3)	11.8 (11.4, 12.2)	11.5 (11.1, 11.9)	11.3 (10.9, 11.7)	11.2 (10.9, 11.6)	11.1 (10.7, 11.5)
Baseline to 2 y	−0.6 (−1.1, −0.1)	−0.6 (−1.1, −0.1)	−0.2 (−0.7, 0.3)	−1.3 (−1.7, −0.8)	−0.9 (−1.4, −0.4)	−0.8 (−1.3, −0.3)
Baseline to 4 y	−0.1 (−0.6, 0.4)	0.4 (−0.1, 0.9)	0.4 (−0.1, 0.9)	−0.4 (−0.9, 0.1)	−0.1 (−0.6, 0.4)	−0.2 (−0.7, 0.3)
*P* for 4-y change within group	0.04	0.001	0.03	0.001	0.001	0.002
*P* for difference in 4-y change between the intervention group and the control group		0.20	0.14	0.30	0.80	0.73

^1^Estimated means (95% CIs) and *P* values are derived from mixed-model analysis; *P* values are adjusted for multiple comparisons using the 2-stage Benjamini-Hochberg procedure (32). Data on aerobic and resistance exercise are based on a 12-mo leisure-time physical activity questionnaire that was modified from the Minnesota Leisure Time Physical Activity Questionnaire. MET-h, metabolic equivalent hours (multiply the MET value for each activity by the hours spent in that activity).

### Changes in cognitive function during 4 y

None of the individual treatments (aerobic exercise, resistance exercise, and diet) had a statistically significant adjusted effect on CERAD-TS over the 4-y intervention period. The adjusted effect of aerobic exercise was 0.2 points (95% CI: −1.1, 1.5 points; ES: 0.02; 95% CI: −0.03, 0.08). The adjusted effect of resistance exercise was 0.5 points (95% CI: −0.8, 1.7 points; ES: 0.05; 95% CI: −0.01, 0.11). The adjusted effect of diet was 0.7 points (95% CI: −0.5, 2.0 points; ES: 0.08; 95% CI: −0.02, 0.14). Diet did not potentiate the effect of aerobic or resistance exercise on CERAD-TS.

There was a trend toward improved adjusted estimated 4-y CERAD-TS in the combined aerobic exercise and diet group compared with the control group (net increase: 1.4 points; 95% CI: 0.1, 2.7; *P* = 0.06) but not in the combined resistance exercise and diet group compared with the control group (*P* = 0.25), with Hedges’ *g* values for ES of 0.15 and 0.09, respectively (**Table 3**).

**TABLE 3:** CERAD-TS at baseline and its change during 2 and 4 y in the 6 study groups^1^

Primary analysis and measurement time	Control (*n* = 234)	Resistance exercise (*n* = 234)	Aerobic exercise (*n* = 234)	Diet (*n* = 235)	Resistance exercise + diet (*n* = 232)	Aerobic exercise + diet (*n* = 232)
CERAD-TS at baseline	82.3 (81.2, 83.4)	82.9 (81.8, 84.0)	82.8 (81.7, 83.9)	82.6 (81.5, 83.3)	82.6 (81.5, 83.7)	82.5 (81.3, 83.6)
Change in CERAD-TS during 2 y	0.7 (−0.3, 1.7)	0.6 (−0.4, 1.6)	0.9 (−0.1, 1.9)	0.4 (−0. 6, 1.4)	0.7 (−0.3, 1.7)	1.2 (0.2, 2.2)
Change in CERAD-TS during 4 y	0.4 (−0.6, 1.5)	0.8 (−0.2, 1.9)	0.5 (−0.5, 1.5)	1.2 (0.2, 2.3)	1.2 (0.2, 2.3)	1.8 (0.8, 2.8)
*P* for 4-y change within the group	0.25	0.15	0.15	0.04	0.05	0.001
*P* for difference in 4-y change between the intervention group and the control group		0.60	0.90	0.34	0.25	0.06
Hedges’ *g*		0.05 (−0.01, 0.11)	0.02 (−0.03, 0.08)	0.08 (−0.02, 0.14)	0.09 (−0.03, 0.15)	0.15 (0.09, 0.21)

^1^The data are from a linear mixed-effects model and denote estimated means (95% CIs) for the CERAD-TS at baseline and changes in CERAD-TS during 2 y and 4 y. *P* values are adjusted for multiple comparisons using the 2-stage Benjamini-Hochberg procedure (32). Hedges’ *g* values are measures of intervention effect after adjustment for age, gender, years of education, symptoms of depression, and waist circumference at baseline. CERAD-TS is a measure of global cognitive performance and denotes the total score of the Consortium to Establish a Registry for Alzheimer's Disease neuropsychological tests, ranging between 0 and 100 with a higher score indicating better performance. CERAD-TS, Consortium to Establish a Registry for Alzheimer's Disease total score.

None of the individual treatments had a statistically significant adjusted effect on CERAD-TS during the first 2 intervention years. Similarly, neither the combination of diet with aerobic exercise nor resistance exercise had statistically significantly larger adjusted effects than the sum of their individual adjusted effects during the first 2 intervention years. There were no statistically significant differences in the adjusted estimated changes in the subtests of CERAD-TS between any intervention group and the control group (see more details in **Supplemental Table 2**).

## Discussion

The DR's EXTRA study is the first long-term RCT on the combined effects of aerobic or resistance exercise and diet intervention on global cognition. The main finding of the study is that regular at least moderate-intensity aerobic exercise combined with a healthy diet but not other combinations of lifestyle interventions showed a trend toward improved global cognition during 4 y in middle-aged and older individuals from a general population. The combination of aerobic exercise and healthy diet or resistance exercise and a healthy diet on global cognition did not differ from that of aerobic exercise, resistance exercise, or diet alone.

Aerobic exercise has been found to improve global cognition compared with a control group in some RCTs among cognitively healthy older individuals (34, 35). In our study, however, there was no difference in the change in global cognition during 4 y between the aerobic exercise group and the control group. Aerobic exercise has often been supervised in other RCTs (34, 35), whereas aerobic exercise was performed without supervision in the DR's EXTRA study because it was considered a realistic approach in our 4-y RCT in a large population sample of middle-aged and older individuals. This difference may partly explain the stronger effect of aerobic exercise on global cognition in earlier studies than in the present study. However, an important observation of our study is that aerobic exercise even without supervision, if combined with a healthy diet, may improve global cognition during 4 y in a general population of middle-aged and older individuals. On the other hand, the lack of any effect of aerobic training alone may be related to the fact that the cohort recruited for this study was already physically active at baseline.

The results of earlier RCTs on the effects of resistance exercise on global cognition are inconsistent (4, 34). A meta-analysis on this topic even concluded that resistance exercise alone can decrease global cognition in cognitively healthy middle-aged and older individuals (34). However, resistance exercise combined with aerobic exercise improved global cognition more than aerobic exercise alone in older individuals without dementia (34). In the present study, participants decreased aerobic exercise by ∼30 min/wk in the resistance exercise group as well as in the combined resistance exercise and diet group, which may weaken the association of resistance exercise on global cognition. In our 4-y RCT in a general population of middle-aged and older individuals, there was a trend toward improved global cognition in the combined resistance exercise and diet group but not in the resistance exercise group. However, there was no difference in the change in cognition between the combined resistance exercise and diet group and the control group. The inconsistent findings of these RCTs may be due to differences in the study populations, the measures of global cognition, and the compliance to resistance exercise.

A healthy diet has been observed to improve global cognition in cognitively healthy older individuals (12), although the number of RCTs addressing this issue is small (13, 14). However, a healthy diet has not improved global cognition in all RCTs in individuals without cognitive impairment (15). We found that global cognition improved during 4 y in the diet group, which followed the Finnish Nutrition Recommendations (20), in a general population of middle-aged and older individuals. However, there was no difference in the 4-y change in global cognition between the diet group and the control group. One explanation for the inconsistent observations of these studies may be that the dietary interventions have been based on different food patterns, such as the Mediterranean diet or the Nordic diet, which makes the comparison of the results difficult. Furthermore, the control group also improved diet quality in the DR's EXTRA study, which may confound the effect of a healthy diet in the diet intervention groups.

The Finnish Geriatric Intervention Study to Prevent Cognitive Impairment and Disability (FINGER) showed that a 2-y multicomponent intervention prevented cognitive decline in middle-aged and older individuals at increased risk of dementia (17). However, the FINGER study did not address the independent and combined effects of aerobic or resistance exercise and diet intervention on cognition because the multicomponent intervention included physical exercise, a healthy diet, cognitive training, stimulating social activity, and cardiovascular risk monitoring and the effects of these interventions were not analyzed separately. Furthermore, the participants of the FINGER study were at increased risk of dementia, whereas the participants of the DR's EXTRA study comprised a general population with a wide range of risk for cognitive decline. The Finnish Diabetes Prevention Study (DPS) among middle-aged individuals with overweight and impaired glucose tolerance showed no difference in cognition after the 13-y follow-up between the combined diet, physical activity, and weight-reduction group and the control group (36). However, it was not possible to investigate the true effect of the lifestyle intervention on cognition in the DPS because cognition was not measured at baseline but only after the 13-y follow-up.

Encouraging individuals to improve and maintain their health behavior is challenging, especially in older individuals (37). The aging process is accompanied by physical, emotional, and psychological changes that often decrease motivation and the possibility to exercise regularly (38). Moreover, it is much more complex and challenging to conduct interventions aimed at improving dietary patterns comprehensively than to carry out single-nutrient interventions (37). If people are not following the lifestyle interventions prescribed, the efficacy of these interventions is expected to be modest. Our compliance analyses based on the goals of the interventions showed that compliance to none of the interventions was optimal. Individuals in the control group may also have improved their lifestyle during the study, because they were given general recommendations on physical activity and diet at baseline due to ethical reasons. Moreover, the participants of lifestyle intervention studies often improve their health behavior even without intervention, which is called the clinical trial effect (38). A systematic review reported increased physical activity in the control groups in ∼30% of exercise intervention studies, particularly in the longer-term and better-quality trials (39). These common phenomena in lifestyle intervention studies may have decreased the observed differences in physical activity, diet, and cognition between the intervention groups and the control group.

The global recommendations for physical activity and a healthy diet (40, 41) were launched a few years before implementing the DR's EXTRA study. This may have resulted in the adoption of a healthy lifestyle in some participants of the DR's EXTRA study. Altogether, 50% of our participants reached the recommendations for aerobic exercise at baseline. Similarly, many participants exceeded the dietary recommendations (20) at baseline. Thus, it is possible that some of the participants had already attained much of the benefits of aerobic exercise and a healthy diet before our RCT, thereby leaving less space for additional improvements.

The strengths of the DR's EXTRA study include the 4-y RCT design and the large population-based random sample of men and women with a wide age range. We found a trend toward improved global cognition when the combined aerobic exercise and diet intervention continued during the last 2 y of follow-up, which highlights the value of the long intervention, as emphasized in systematic reviews and meta-analyses (5–10, 18). The large number of participants in each study group provided us with sufficient power for the statistical analyses, which has not been the case in many other studies (18). We used the ITT principle, which is the recommended approach in the statistical analyses of RCTs (18). We also reported compliance to the interventions and the number of dropouts in each group, as recommended (18). We used valid and commonly used assessments of diet and physical activity in the evaluation of achieving the dietary goals and assessing compliance to the interventions (42). We acknowledge that underreporting of the consumption of certain foods is commonly related to the assessment of diet and it is difficult to recall physical activities over the past year (23). However, the inaccuracy of the assessment of diet and physical activity is expected to be similar in all study groups.

We used the CERAD tests to assess global cognition because they are feasible in large study populations, have good interrater and test–retest reliability (21), and provide a more reliable picture of the global cognition than the widely used MMSE (43). Two years between CERAD tests has been found to be a sufficiently long interval to minimize a possible learning effect (44). However, there was no decrease in CERAD-TS in any of the study groups during the 4-y follow-up, which could be partly due to the learning effect. Study nurses were trained by a neuropsychologist. However, we cannot totally exclude the possibility of between-observer variation in CERAD-TS measurements and cannot totally exclude the possibility that some participants might have had incipient cognitive dysfunction already at baseline.

In summary, the DR's EXTRA study provides additional information on the effects of lifestyle interventions on cognition beyond the evidence from earlier RCTs by suggesting that the combination of aerobic exercise and a healthy diet may be needed to improve global cognition in a general population of middle-aged and older individuals. These observations are important from a public health perspective, because they suggest that even small effects of long-term and multifaceted lifestyle interventions may postpone cognitive impairment in older individuals (45). These findings could be used to encourage people to increase physical activity and improve diet and for health care professionals to emphasize these lifestyle changes for the prevention of dementia.

## Supplementary Material

nqab018_Supplemental_File

## Data Availability

Data described in the manuscript, code book, and analytic code will not be made available because ethical permissions do not allow it.

## Notes

This study was supported by grants from the Ministry of Education and Culture of Finland (722 and 627; 2004-2010); Academy of Finland (102318, 104943, 123885, 121119); the European Commission FP6 Integrated Project (EXGENESIS), LSHM-CT-2004-005272; City of Kuopio; Juho Vainio Foundation; Finnish Diabetes Association; Finnish Foundation for Cardiovascular Research; Kuopio University Hospital; Päivikki and Sakari Sohlberg Foundation; and the Social Insurance Institution of Finland (4/26/2010). The sponsors of the study played no part in the preparation of this article.

Supplemental Figure 1 and Supplemental Tables 1 and 2 are available from the “Supplementary data” link in the online posting of the article and from the same link in the online table of contents at https://academic.oup.com/ajcn/.

Abbreviations used: BIC, Bayesian information criterion; CERAD, Consortium to Establish a Registry for Alzheimer's Disease; CERAD-TS, CERAD total score; DPS, Diabetes Prevention Study; DR's EXTRA, Dose-Responses to Exercise Training; E%, % of energy; ES, effect size; FINGER, Finnish Geriatric Intervention Study to Prevent Cognitive Impairment and Disability; IMT, intima-media thickness; ITT, intention-to-treat; MET, metabolic equivalent; MMSE, Mini-Mental State Examination; RCT, randomized controlled trial; RM, repetition maximum; VIF, variance inflation factor.
