# Supplementary material for: Exercise, diet, and cognition in a 4-year randomized controlled trial: Dose-Responses to Exercise Training (DR's EXTRA)
Source: Am J Clin Nutr. 2021 Mar 19;113(6):1428–39. doi: 10.1093/ajcn/nqab018 (PMC8244125; doi:10.1093/ajcn/nqab018)
Supplement: nqab018_Supplemental_File [file nqab018_supplemental_file.docx]

**Exercise, diet and cognition in a 4-year randomized controlled trial - DR’s EXTRA**

Pirjo Komulainen “Online Supplementary Material”

**Supplementary table 1** Supplementary Material

The predefined exclusion criteria that prohibit engagement in exercise intervention

or the assessments, as judged by a physician

| Unstable angina pectoris |
| --- |
| Myocardial infarction less than 2 months prior to maximal exercise test |
| Bypass operation less than 12 months prior to maximal exercise test |
| Percutaneous coronary intervention less than 3 months prior to maximal exercise test |
| Tachycardia (atrial or ventricular) |
| Atrioventricular block (II-III degree) |
| Endo-, myo- and/or pericarditis |
| Serious aortic stenosis |
| Decompensated cardiac insufficiency |
| Acute pulmonary embolism |
| Acute infection |
| Renal insufficiency |
| Untreated hypo- or hyperthyreosis |
| Physically disabled on maximal exercise test |
| Mentally disabled on maximal exercise test |

**Supplementary Table 2 S**upplementary material

Adjusted estimated changes in individual subtests of the CERAD total score during four years

| **Subtests of CERAD-TS**  **and measurement time** | **Control**  n=234 | **Resistance**  n=234 | **Aerobic**  n=234 | **Diet**  n=235 | **Resistance+Diet**  n=232 | **Aerobic+Diet**  n=232 |
| --- | --- | --- | --- | --- | --- | --- |
| Verbal fluency, score  baseline  2-year change  4-year change  p for 4-year change within group | 23.9 (23.2, 24.7)  0.6 (-0.3, 1.4)  0.4 (-0.5,o 1.2)  0.34 | 24.3 (23.5, 25.0)  0.7 (-0.2, 1.5)  0.5 (-0.4, 1.3)  0.25 | 24.6 (23.8, 25.4)  0.6 (-0.2, 1.5)  0.2 (-0.7, 1.1)  0.25 | 23.6 (22.9, 24.4)  0.3 (-0.6, 1.1)  0.1 (-0.8, 1.0)  0.80 | 24.2 (23.4, 25.0)  1.0 (0.1, 1.8)  0.6 (-0.3, 1.5)  0.06 | 23.8 (23.0, 24.6)  1.2 (0.3, 2.0)  1.1 (0.2, 2.0)  0.01 |
| p for difference in 4-year change between the intervention group and the control group |  | 0.90 | 0.80 | 0.60 | 0.60 | 0.25 |
| Modified Boston naming, score  baseline  2-year change  4-year change  p for 4-year change within group | 13.0 (12.8, 13.3)  0.3 (0.1, 0.5)  0.2 (0.03, 0.5)  0.02 | 13.2 (13.0, 13.4)  0.2 (-0.03, 0.4)  0.1 (-0.1, 0.4)  0.15 | 13.4 (13.2, 13.6)  0.1 (-0.1, 0.3)  0.2 (-0.04, 0.4)  0.25 | 13.1 (12.9, 13.3)  0.2 (0.01, 0.4)  0.4 (0.2, 0.7)  0.001 | 13. 2 (13.0, 13.4)  0.1 (-0.1, 0.3)  0.2 (-0.02, 0.4)  0.90 | 13.3 (13.1, 13.5)  0.2 (-0.03, 0.4)  0.3 (0.1, 0.5)  0.01 |
| p for difference in 4-year change between the intervention group and the control group |  | 0.42 | 0.50 | 0.15 | 0.70 | 0.60 |
| Word list memory, score  baseline  2-year change  4-year change  p for 4-year change within group | 21.8 (21.4, 22.3)  0.1 (-0.3, 0.6)  0.2 (-0.3, 0.7)  0.50 | 21.8 (21.3, 22.2)  0.1 (-0.4, 0.6)  0.6 (0.1, 1.1)  0.04 | 21.8 (21.3, 22.2)  0.2 (-0.3, 0.7)  0.4 (-0.1, 0.9)  0.25 | 21.8 (21.4, 22.3)  0.2 (-0.3, 0.7)  0.5 (-0.04, 1.00)  0.15 | 22.0 (21.5, 22.4)  -0.1 (-0.6, 0.4)  0.5 (0.02, 1.1)  0.04 | 21.7 (21.3, 22.1)  0.4 (-0.1, 0.9)  0.6 (0.1, 1.1)  0.05 |
| p for difference in 4-year change between the intervention group and the control group |  | 0.34 | 0.60 | 0.50 | 0.42 | 0.25 |

| Constructional praxis, score  baseline  2-year change  4-year change  p for 4-year change within group | 9.6 (9.4, 9.7)  -0.1 (-0.3, 0.1)  -0.2 (-0.4, 0.01)  0.15 | 9.7 (9.5, 9.8)  -0.3 (-0.4, -0.1)  -0.3 (-0.5, -0.1)  0.004 | 9.5 (9.4, 9.7)  -0.1 (-0.2, 0.1)  -0.2 (-0.4, -0.1)  0.04 | 9.8 (9.6, 9.9)  -0.2 (-0.4, 0.001)  -0.3 (-0.5, -0.1)  0.01 | 9.6 (9.5, 9.7)  -0.1 (-0.2, 0.1)  -0.2 (-0.4, -0.03)  0.06 | 9.6 (9.5, 9.8)  -0.1 (-0.3, 0.1)  -0.2 (-0.4, 0.0)  0.15 |
| --- | --- | --- | --- | --- | --- | --- |
| p for difference in 4-year change between the intervention group and the control group |  | 0.42 | 0.60 | 0.42 | 0.70 | 0.90 |
| Word list recall, score  baseline  2-year change  4-year change  p for 4-year change within group | 7.1 (7.0, 7.3)  0.2 (-0.1, 0.4)  0.2 (-0.01, 0.5)  0.15 | 7.2 (7.0, 7.4)  0.2 (-0.04, 0.5)  0.3 (-0.01, 0.5)  0.08 | 7.1 (6.8, 7.3)  0.3 (0.1, 0.6)  0.3 (0.02, 0.5)  0.02 | 7.3 (7.0, 7.5)  0.04 (-0.2, 0.3)  0.3 (0.1, 0.6)  0.02 | 7.2 (7.0, 7.4)  0.1 (-0.1, 0.4)  0.3 (0.02, 0.5)  0.08 | 7.2 (7.0, 7.4)  0.1 (-0.1, 0.4)  0.3 (0.1, 0.6)  0.02 |
| p for difference in 4-year change between the intervention group and the control group |  | 0.99 | 0.80 | 0.60 | 0.80 | 0.50 |
| Word list recognition, score  baseline  2-year change  4-year change  p for 4-year change within group | 9.4 (9.3, 9.5)  0.2 (0.1, 0.4)  0.2 (0.004, 0.3)  0.01 | 9.5 (9.4, 9.6)  0.2 (0.03, 0.3)  0.2 (0.03, 0.4)  0.02 | 9.3 (9.2, 9.4)  0.2 (0.04, 0.4)  0.1 (-0.03, 0.30)  0.01 | 9.5 (9.4, 9.6)  0.1 (-0.1, 0.2)  0.2 (0.01, 0.3)  0.08 | 9.4 (9.3, 9.5)  0.1 (-0.1, 0.3)  0.1 (-0.03, 0.3)  0.15 | 9.4 (9.3, 9.6)  0.2 (0.02, 0.3)  0.3 (0.1, 0.4)  0.003 |
| p for difference in 4-year change between the intervention group and the control group |  | 0.80 | 0.80 | 0.99 | 0.70 | 0.34 |

Adjusted estimated means (95% confidence intervals) and p-values are derived from mixed model analysis adjusted for age, sex, education years, symptoms of depression and waist circumference at baseline. P-values are adjusted for multiple comparisons using the two stage Benjamini-Hochberg procedure. CERAD-TS denotes total score of the Consortium to Establish a Registry for Alzheimer's Disease neuropsychological tests, range from 0 to 100 with higher score indicating a better performance.

**Supplementary Figure 1.** Supplementary Material

Study design and recruitment of the participants

**1479**

Participated in the baseline examinations in 2005-2006

April 5, 2005 - October 4, 2006

**3000**

A random sample of men and women aged 55-74 years from

city of Kuopio invited to the study in 2002

283 Ineligible

53 Death

159 Impaired health

(7 dementia)

71 Moved elsewhere

1028 Declined or other

210 No response

69 Excluded

0 Death

48 Impaired physical

health (1 dementia)

2 Moved elsewhere

19 Declined or other

**1410**

**Randomized in 2005-2006**

**234**

Aerobic exercise

**234**

Resistance+Diet

**234**

Aerobic+Diet

**236**

Resistance exercise

**236**

Diet

**236**

Control

**1199 individuals participated in four-year examinations in October 5, 2009 - March 15, 2011**

**202**

Aerobic exercise

Lost 14% (32)

**192**

Resistance+Diet

Lost 18% (42)

**207**

Aerobic+Diet

Lost 12% (27)

**201**

Resistance exercise

Lost 15% (35)

**196**

Diet

Lost 17% (40)

**201**

Control

Lost 15% (35)

**1292 individuals participated in two-year examinations in May 22, 2007 - December 17, 2008**

**217**

Aerobic exercise

**212**

Resistance+Diet

**218**

Aerobic+Diet

**210**

Resistance exercise

**216**

Diet

**219**

Control

0 Death

4 Disease

0 Moved elsewhere

12 Personal reason

0 Unknown reason

2 Death

6 Disease

0 Moved elsewhere

7 Personal reason

2 Unknown reason

3 Death

4 Disease

2 Moved elsewhere

15 Personal reason

2 Unknown reason

4 Death

4 Disease

3 Moved elsewhere

10 Personal reason

1 Unknown reason

7 Death

2 Disease

0 Moved elsewhere

11 Personal reason

0 Unknown reason

0 Death

4 Disease

1 Moved elsewhere

10 Personal reason

2 Unknown reason

*Lost to follow-up before two-year examinations (8%)*

5 Death

3 Disease

1 Moved elsewhere

2 Personal reason

0 Unknown reason

1 Death

6 Disease

1 Moved elsewhere

10 Personal reason

0 Unknown reason

1 Death

6 Disease

0 Moved elsewhere

2 Personal reason

0 Unknown reason

5 Death

7 Disease

1 Moved elsewhere

6 Personal reason

1 Unknown reason

2 Death

8 Disease

1 Moved elsewhere

7 Personal reason

2 Unknown reason

1 Death

6 Disease

0 Moved elsewhere

6 Personal reason

2 Unknown reason

*Lost to follow-up before four-year examinations (15%)*

**1401 individuals included in intention-to-treat analyses**

**234**

Aerobic exercise

**232**

Resistance+Diet

**232**

Aerobic+Diet

**234**

Resistance exercise

**235**

Diet

**234**

Control
